# Molecular neuropathology of the synapse in sheep with CLN5 Batten disease

**DOI:** 10.1002/brb3.401

**Published:** 2015-10-09

**Authors:** Inês S. Amorim, Nadia L. Mitchell, David N. Palmer, Stephen J. Sawiak, Roger Mason, Thomas M. Wishart, Thomas H. Gillingwater

**Affiliations:** ^1^ Centre for Integrative PhysiologyUniversity of EdinburghHugh Robson BuildingEdinburghUK; ^2^ Euan MacDonald Centre for Motor Neurone Disease ResearchUniversity of EdinburghHugh Robson BuildingEdinburghUK; ^3^ Department of Molecular BiosciencesFaculty of Agricultural and Life Sciences and Batten Animal Research NetworkLincoln UniversityChristchurchNew Zealand; ^4^ Department of Physiology, Development and NeuroscienceUniversity of CambridgeDowning StreetCambridgeUK; ^5^ Wolfson Brain Imaging CentreUniversity of CambridgeBox 65 Addenbrooke's HospitalHills RoadCambridgeUK; ^6^ Division of NeurobiologyThe Roslin Institute and Royal (Dick) School of Veterinary StudiesUniversity of EdinburghEdinburghUK

**Keywords:** Animal model, lysosomal storage disorders, neuronal ceroid lipofuscinoses, neurodegeneration, neuronal ceroid lipofuscinosis, sheep, synapse, synaptic vulnerability

## Abstract

**Aims:**

Synapses represent a major pathological target across a broad range of neurodegenerative conditions. Recent studies addressing molecular mechanisms regulating synaptic vulnerability and degeneration have relied heavily on invertebrate and mouse models. Whether similar molecular neuropathological changes underpin synaptic breakdown in large animal models and in human patients with neurodegenerative disease remains unclear. We therefore investigated whether molecular regulators of synaptic pathophysiology, previously identified in Drosophila and mouse models, are similarly present and modified in the brain of sheep with CLN5 Batten disease.

**Methods:**

Gross neuropathological analysis of CLN5 Batten disease sheep and controls was used alongside postmortem MRI imaging to identify affected brain regions. Synaptosome preparations were then generated and quantitative fluorescent Western blotting used to determine and compare levels of synaptic proteins.

**Results:**

The cortex was particularly affected by regional neurodegeneration and synaptic loss in CLN5 sheep, whilst the cerebellum was relatively spared. Quantitative assessment of the protein content of synaptosome preparations revealed significant changes in levels of seven out of eight synaptic neurodegeneration proteins investigated in the motor cortex, but not cerebellum, of CLN5 sheep (*α*‐synuclein, CSP‐*α*, neurofascin, ROCK2, calretinin, SIRT2, and UBR4).

**Conclusions:**

Synaptic pathology is a robust correlate of region‐specific neurodegeneration in the brain of CLN5 sheep, driven by molecular pathways similar to those reported in Drosophila and rodent models. Thus, large animal models, such as sheep, represent ideal translational systems to develop and test therapeutics aimed at delaying or halting synaptic pathology for a range of human neurodegenerative conditions.

## Introduction

Progressive neurological conditions such as Alzheimer's disease, Parkinson's disease, Huntington's disease, and some lysosomal storage disorders, including the neuronal ceroid lipofuscinoses (NCLs; Batten disease; http://www.ucl.ac.uk/ncl (Kousi et al. 2012)), are a genetically, pathologically and clinically heterogeneous group of diseases. Recent molecular and pathological evidence points to a shared feature of many such diseases: a critical vulnerability of synapses in affected brain regions (Wishart et al. 2006; Gillingwater and Wishart 2013). Thus, dysfunction and breakdown of synaptic connections is often one of the best neuropathological correlates of a patient's functional decline. For example, the onset of synaptic pathology strongly correlates with the onset of neurological symptoms in both Alzheimer's disease (Davies et al. 1987; Terry et al. 1991) and Huntington's disease (Li et al. 2003). Similarly, synaptic dysfunction and degeneration is an early event occurring in affected brain regions during many genetically distinct forms of Batten disease (NCL) (Virmani et al. 2005; Luiro et al. 2006; Partanen et al. 2008; Kielar et al. 2009; Koch et al. 2011).

That synaptic pathology represents a common cellular event across numerous neurological disorders raises the possibility of shared molecular mechanisms regulating synaptic pathophysiology that are independent of the neurodegenerative “trigger”. This hypothesis is supported by several lines of experimental evidence. For example, a spontaneous genetic mutation that robustly delays synaptic degeneration (known as *Wld* ^*S*^) can ameliorate neurodegeneration across a range of conditions, including some forms of motor neuron disease (Ferri et al. 2003), Parkinson's disease (Sajadi et al. 2004) and following global cerebral ischemia (Gillingwater et al. 2004) or traumatic brain injury (Gillingwater et al. 2006). Furthermore, at the molecular level, a number of individual synaptic proteins that can modulate synaptic vulnerability and degeneration in response to a variety of pathological stimuli have recently been identified, including CSP alpha/DNAJC5 (Fernández‐Chacón et al. 2004; García‐Junco‐Clemente et al. 2010; Nosková et al. 2011; Wishart et al. 2012; Kashyap et al. 2014).

Our growing awareness of the mechanisms underlying synaptic pathophysiology raises the possibility that targeted synapto‐protective therapies could be developed for the treatment of a variety of neurodegenerative conditions. One major issue facing such translational neurodegeneration research, however, is the current overreliance on small animal models, such as mice, that do not fully replicate many size‐ and age‐related aspects of the human nervous system (Aigner et al. 2010; Morton and Howland 2013; Pouladi et al. 2013; Dolezalova et al. 2014). The growing availability of large animal models of neurodegeneration, including sheep and pigs, offers one solution to this problem, but it has yet to be established if the mechanisms underlying synaptic pathophysiology that have been identified in rodent and *Drosophila* models are conserved in larger species.

Although genetically modified large animal models of neurodegeneration are becoming more commonplace (Aigner et al. 2010; Morton and Howland 2013; Dolezalova et al. 2014), there are also a number of human neurodegenerative conditions where there is an equivalent endogenous condition in a large animal species. Batten disease is exceptional in this regard, with research on naturally occurring large animal models having led the way for five decades (Palmer et al. 2011; Bond et al. 2013). Batten disease often refers to a group of fatal neurodegenerative disorders that mainly begin in childhood, representing the most common group of childhood neurodegenerative diseases, also collectively known as the neuronal ceroid lipofuscinoses (NCLs) (Palmer et al. 2011; Bond et al. 2013). The sheep used in this study have a late infantile variant CLN5 form of Batten disease. A number of naturally occurring large animal forms of CLN5 Batten disease have been described, including two in dogs (Melville et al. 2005; Gilliam et al. 2015), a form in cattle (Houweling et al. 2006), and this well‐developed form in New Zealand Borderdale sheep, which have a c.571 + 1G>A splice site mutation in the *CLN5* gene that causes disease (Jolly et al. 2002; Frugier et al. 2008; Palmer et al. 2015).This mutation leads to the loss of a 5′ splice donor site between exon and intron 3, resulting in excision of exon 3, a downstream frame shift and premature termination of translation.

The major neuropathological features of CLN5 Batten sheep closely mirror those of human CLN5 Batten disease (a variant late‐infantile Batten disease) caused by mutations in the human *CLN5* gene (Jolly et al. 2002; Frugier et al. 2008; Palmer et al. 2015; Perentos et al. 2015). As with many other forms of Batten disease, mutations in the *CLN5* gene have been shown to trigger early synaptic pathology in the affected brain regions of mouse models (von Schantz et al. 2009). We therefore set out to establish the feasibility of isolating synapses from the brain of sheep with Batten disease in order to test whether known molecular regulators of synaptic pathophysiology, previously identified in *Drosophila* and mouse models, are similarly present and modified in the brain of sheep with CLN5 Batten disease.

## Materials and Methods

### Ethics statement

All animal procedures were carried out in accordance with NIH guidelines and the New Zealand Animal Welfare Act 1999 as approved by the Lincoln University Animal Ethics committee. Experimental procedures performed in Cambridge were conducted in accordance with the UK Animals (Scientific Procedures) Act 1986 and were approved by a University of Cambridge internal ethical review board.

### Animals

CLN5 sheep and heterozygous controls were born, raised, and diagnosed from a flock of Borderdale sheep at Lincoln University (New Zealand) (Frugier et al. 2008; Palmer et al. 2015). Heterozygous sheep were chosen as controls because they do not develop any signs of disease (based on clinical and postmortem observations of over a hundred breeding ewes kept for an average of seven years) but otherwise share their genetic background with the homozygous sheep.

Sheep for gross neuropathological analyses were sacrificed at the Lincoln University by exsanguination, and the head perfusion‐fixed via one of the carotid arteries with 10% formalin in 0.9% NaCl, pH7.4, after the blood was first cleared with 0.9% NaCl (37°C). The brains were bisected at the sagittal midline, fixed for a further 7 days in 10% formalin, equilibrated in cryoprotective solution (10% ethylene glycol, 20% sucrose in 0.9% NaCl), and stored frozen at −80°C until they were sectioned.

Cohorts of heterozygous controls and presymptomatic CLN5 sheep for MRI and molecular analyses were shipped by air to the University of Cambridge (U.K.) where they were housed in a barn with windows and supplementary additional artificial light (from 6 am to 6 pm). All animals had free access to hay feed and water, with additional pellet supplements provided every morning. Sheep were sacrificed following an i.v. overdose of barbiturate‐based anesthetic. Selected brain regions were flash‐frozen by submersion in liquid nitrogen before being stored at −80°C until ready for shipping to Edinburgh. Postmortem delay from death to samples being frozen was 15–20 min per animal. Tissue was obtained from eight 20–24‐month‐old CLN5 female sheep, and five heterozygous controls, four females and one castrated male, of comparable age.

Young adult C57BL/6 (wild‐type) mice were obtained from in‐house breeding stocks at the University of Edinburgh. Mice were killed by inhalation overdose of Isoflurane, in accordance with the guidance and rules of the UK Home Office. Brains were immediately dissected and used for synaptosome preparation.

### Gross pathology

Cryopreserved perfusion fixed frozen brain hemispheres were serially sectioned at 50 μm on a sliding microtome (Microm, Germany) and the sections mounted on slides, stained with Nissl, then coverslipped (Oswald et al. 2005). Images were captured using a Nikon Eclipse 50i model microscope and cortical thicknesses in three animals of each genotype at 24 months of age were determined using NIS‐Elements Software (Nikon Instruments Inc., Melville, NY). At least 25 measurements were made for each animal from the pial surface to the white matter (cortical layers I–VI) in the primary motor cortex and from the pial surface to the granular boundary with the white matter in the anterior lobe of the cerebellum.

### Magnetic resonance imaging

To compare the morphology of CLN5 Batten disease sheep against heterogeneous gene carriers, we scanned each using magnetic resonance imaging (MRI). A 4.7T Bruker BioSpec 47/40 was used to image brains from sheep that had been euthanized and then perfusion fixed in 4% paraformaldehyde in saline. Heads were trimmed to remove the jaw and orbits but the brains were not removed from the cranium to preserve the cortical surface.

Brains were imaged using a rapid acquisition with relaxation enhancement protocol with a field of view of 128 × 85.2 × 66.6 mm^3^ with a matrix of 384 × 256 × 200 with twofold anti‐aliasing in all three directions to provide an isotropic resolution of 333 μm. The repetition time was 1.2 s with an affective echo time of 50 ms with an echo train length of five and a bandwidth of 50 kHz with a total scan time of 13.5 h per brain. For comparison, reconstructed images were rigidly registered together, using SPM12 (Wellcome Trust Centre for Neuroimaging, University College London) and the SPMMouse toolbox (Sawiak et al. 2013).

### Synaptosome preparation

Synaptosomes were prepared from fresh mouse whole brain tissue and flash‐frozen sheep brain tissue. Frozen samples were briefly incubated in a water bath at 37°C to ensure they were completely thawed before proceeding. Due to the size of the sheep brain regions and the necessity to use relatively small, and therefore heterogeneous, samples for the efficient preparation of synaptosomes, two independent synaptosome samples were prepared and analyzed for each animal.

Brain samples were homogenized in ice‐cold sucrose buffer (0.32M sucrose, 5mM Tris‐HCl, 1mM EDTA, pH 7.4), using a glass plunger and centrifuged for 10 min at 900g and 4°C. The supernatant was collected and the pellet resuspended in sucrose buffer and centrifuged as before. The supernatant was combined with the supernatant from the initial spin and centrifuged at 20,000*g* for 15 min to pellet out crude synaptosomes. The supernatant was discarded and the synaptosomes stored at −80°C until required.

### Quantitative fluorescent Western blot analysis

Quantitative Western blotting was used to determine relative protein levels, as previously described (Roche et al. 2014; Wishart et al. 2014). Briefly, all proteins extracted from synaptosome and whole tissue preparations were homogenized and extracted in RIPA buffer (ThermoScientific, Paisley, U.K.) with a protease inhibitor cocktail (ThermoScientific) and the protein concentration determined by BCA assay (ThermoScientific). The extracted protein was then separated using SDS–polyacrylamide gel electrophoresis (Precast NuPage 4–12% Bis‐Tris gradient gels [Invitrogen, Paisley, U.K.]) before being transferred to PVDF membrane, using the iBlot semi‐dry blotting system (Invitrogen). PVDF membranes were then exposed to standard blocking solution (LI‐COR, Biosciences, Cambridge, U.K.) for 30 min at room temperature. Quantitative fluorescent Western blots were performed using primary antibodies against *α*‐synuclein (1:1000, Santa Cruz), *β*‐actin (1:5000, Abcam), calretinin (1:1000, Swant), CSP‐*α* (1:1000, Abcam), CNPase (1:250, Abcam), HDAC2 (1:250, Active Motif), neurofascin (1:5000, Abcam), ROCK2 (1:250, Abcam), SIRT2 (1:500, Abcam), SV2 (1:5000, Development Studies Hybridoma Bank), UBR4 (1:1000, Abcam). All primary antibodies were diluted in blocking solution with 0.1% Tween‐20 (Sigma, Dorset, U.K.) and were applied overnight at 4°C. Following several washes in PBS, Odyssey secondary antibodies (goat anti‐rabbit IRDye 680 or donkey anti‐mouse IRDye 800, LI‐COR Biosciences) were diluted in blocking solution with 0.1% Tween‐20 (Sigma) and added to membranes for 1 h (room temperature). Blots were washed again in PBS (3 × 10 min washes) before being dried and imaged, using an Odyssey Infrared Imaging System (LI‐COR, Biosciences). Each blot was scanned independently and the intensity of each fluorescent band was measured in triplicate, in order to minimize user variability, using the Image Studio software (LI‐COR, Biosciences).

### Statistical analysis

Data were collected into Microsoft Excel spreadsheets and analyzed, using GraphPad Prism software. All data are reported as mean ± SEM. Individual statistical tests used are detailed in figure legends. Statistical significance was considered to be *P ≤ *0.05 for all analyses. Figures were created using Adobe Photoshop.

## Results

### Identification of brain regions that undergo neurodegeneration in CLN5 sheep brain

**Figure 1:**
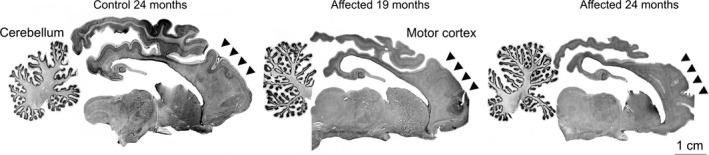
Gross cortical atrophy in CLN5 sheep brains. Nissl‐stained sections showed marked atrophy of the cortical mantle in affected sheep at 19 months of age, which was more pronounced by 24 months of age. In contrast, the cerebellum and subcortical structures were relatively spared.

**Figure 2:**
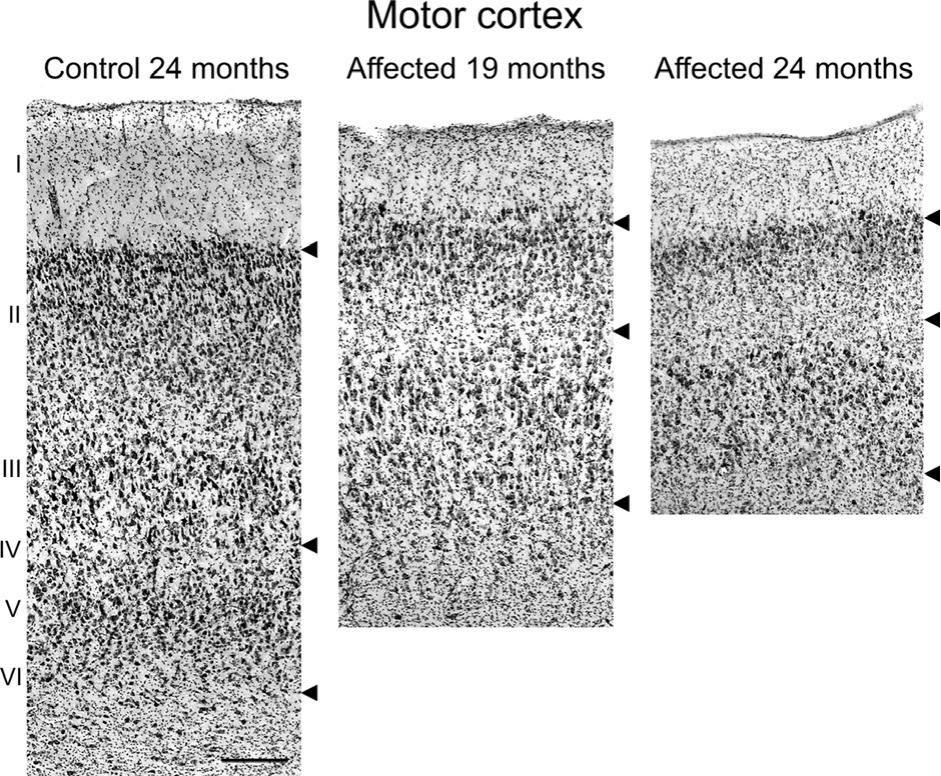
Atrophy of motor cortex in CLN5 sheep brains. Microscopic comparison of the motor cortex of Nissl‐stained sections (shown in Fig. 1). Upper arrows mark the layer I/II boundary, the middle arrows indicate layer IV, and the lower arrow marks the layer VI/white matter boundary. Scale bar 200 μm.

Regional variations in neurodegeneration in CLN5 Batten disease sheep brain were initially investigated to identify an area of the brain subject to substantial neurodegeneration at the late stage disease, as well as an area where there is very little, if any, neurodegeneration. Gross neuropathological changes were analyzed in complete sagittal sections of late stage ovine CLN5 Batten disease. Atrophy of all cortical regions was obvious macroscopically at 24 months, however, both subcortical nuclei and the cerebellum of CLN5 brains retained a normal appearance (Fig. 1). Nissl‐stained sections were analyzed for cortical thickness and cytoarchitectural changes in different brain regions. Cortical atrophy was most severe, in descending order, in the primary visual, parieto‐occipital and motor cortex regions of the CLN5 sheep brain, with progressive thinning of the neuronal layers, particularly the upper layers (II–III), and by 24 months of age few cortical neurons were left (Fig. 2). At this age, the cortical thickness of the primary motor area of CLN5 animals was only 56% of that observed in normal controls (Table 1; Fig. 2). In contrast, no disease‐related cytoarchitectural differences were apparent in the cerebellum between control and CLN5 animals (Fig. 1), and there was no detectable difference in cerebellar cortical thicknesses (Table 1).

**Table 1:** Cortical thickness measurements from 24‐month‐old normal control and CLN5 Batten sheep brains

Layers	Mean thickness (μm ± SEM)^a^		*P* value^b^
	Normal control	CLN5 affected	
Cerebellar cortex	502 ± 10	493 ± 11	NS
Motor cortex (I–VI)	1692 ± 45	940 ± 21	≤0.001

a Measurements from three animals per group and >25 measurements per brain/region.

b Student's *t* test.

**Figure 3:**
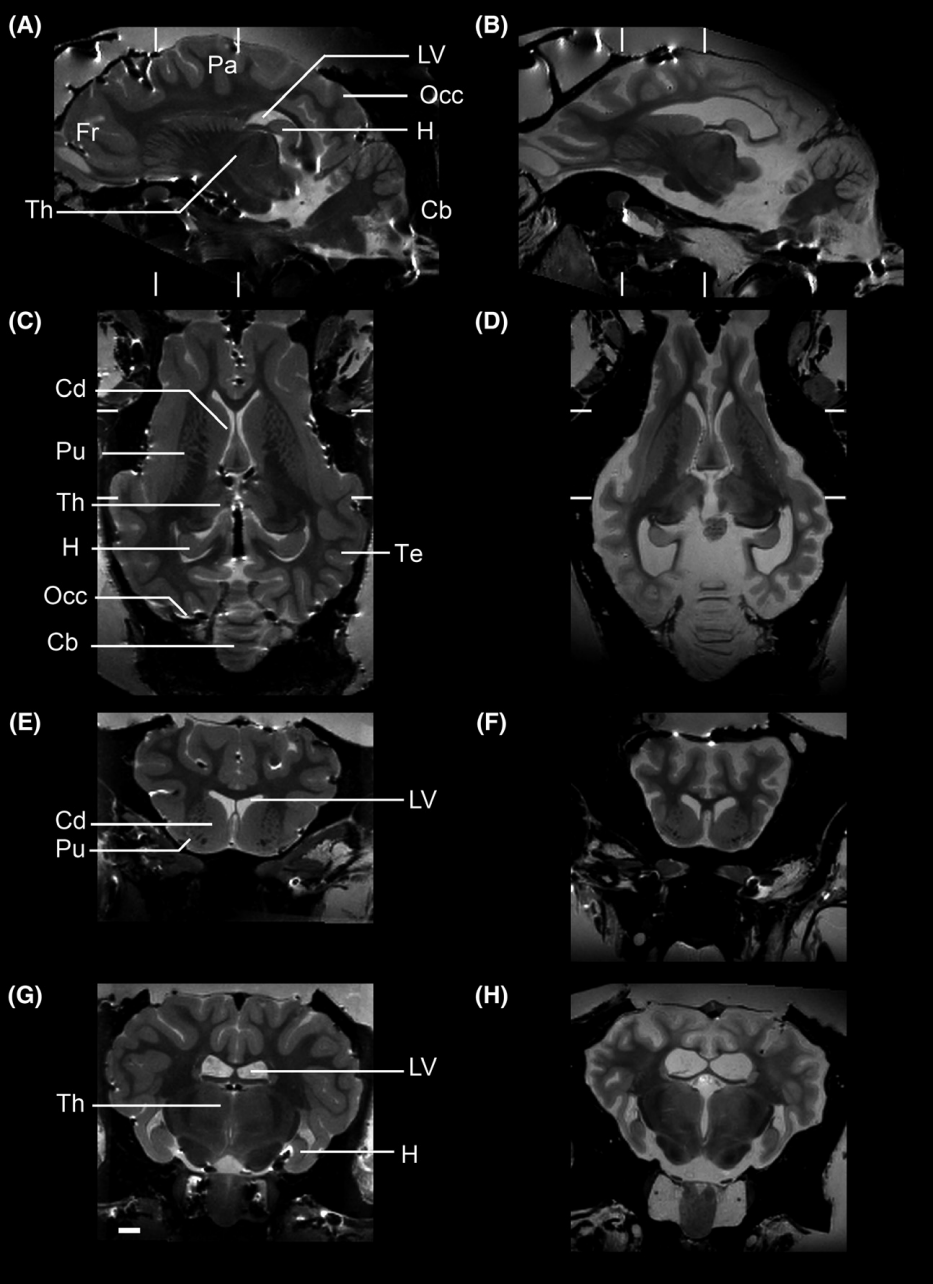
MRI analyses of CLN5 Batten sheep brain confirms targeting of the cortex and sparing of the cerebellum. Representative MR images of control (A, C, E, G) and CLN5 sheep (B, D, F, H). Sections are shown in sagittal (A–B), horizontal (C–D) and two coronal cuts (E–F and G–H at the levels marked in A–D). The scale bar is 2 cm. Label abbreviations: Fr frontal lobe; Pa parietal lobe; Occ occipital lobe; Te temporal lobe; LV lateral ventricle, Th thalamus, H hippocampus; Cb cerebellum; Cd caudate; Pu putamen.

Neuropathological changes in CLN5 sheep were also studied by MRI of perfusion fixed brains in situ. Representative MRI images of control (heterozygous) and CLN5 Batten disease sheep are shown in Figure 3. Compared to brains of control sheep, brains from CLN5 Batten disease sheep had the same striking reduction in neocortical volume, with relative sparing of subcortical structures and the cerebellum.

Taken together, these neuropathological studies confirmed that CLN5 deficiency in sheep affects brain areas in a differential manner, with the cerebellum being relatively spared from disease‐related neurodegeneration and the cortex being severely affected. Since the motor cortex was grossly degenerate and easily accessible, tissue was harvested from this region for subsequent biochemical comparison with cerebellar tissue.

### Synapse loss in affected regions of CLN5 Batten sheep brain

**Figure 4:**
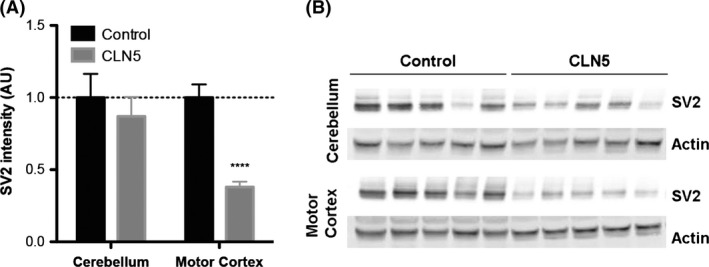
Loss of synapses in affected brain regions from CLN5 Batten disease sheep. A. Quantitative fluorescent Western blotting in preparations of whole tissue revealed relatively stable levels of SV2, a core synaptic protein, in the cerebellum of control and CLN5 sheep. In contrast, in the motor cortex of CLN5 Batten disease sheep, the levels of SV2 are reduced by 60%, indicating there is severe synapse loss restricted to this brain area of the Batten sheep. (*N* = 5 animals, *n* = 10 distinct tissue samples from control sheep; *N* = 8, *n* = 16 from CLN5 sheep; ****P < 0.0001, unpaired two‐tailed *t*‐test). B. Representative fluorescent Western blots (*N* = 5 animals per genotype) showing a consistent decrease in SV2 levels in the motor cortex of CLN5 Batten disease sheep when compared to controls. Note the relatively stable levels of SV2 in the cerebellum of CLN5 sheep. Actin was used as loading control.

Given that synapse loss has been identified as a feature of CLN5 pathology in mouse models (von Schantz et al. 2009), we wanted to verify whether synapses were also being targeted in the CLN5 Batten sheep. Quantitative fluorescent Western blotting was initially used to measure the synaptic protein SV2 in whole tissue samples from a brain region unaffected by neurodegeneration (the cerebellum). SV2 levels remained unaltered in the cerebellum of CLN5 sheep compared to controls, confirming the low vulnerability of synapses in this specific region of the brain (Fig. 4). In contrast, SV2 levels were significantly reduced in a region where neurodegeneration was significant (the motor cortex) in CLN5 Batten sheep, indicative of a substantial loss of synapses (Fig. 4).

### Isolation of enriched synaptic preparations (synaptosomes) from CLN5 Batten sheep brain

**Figure 5:**
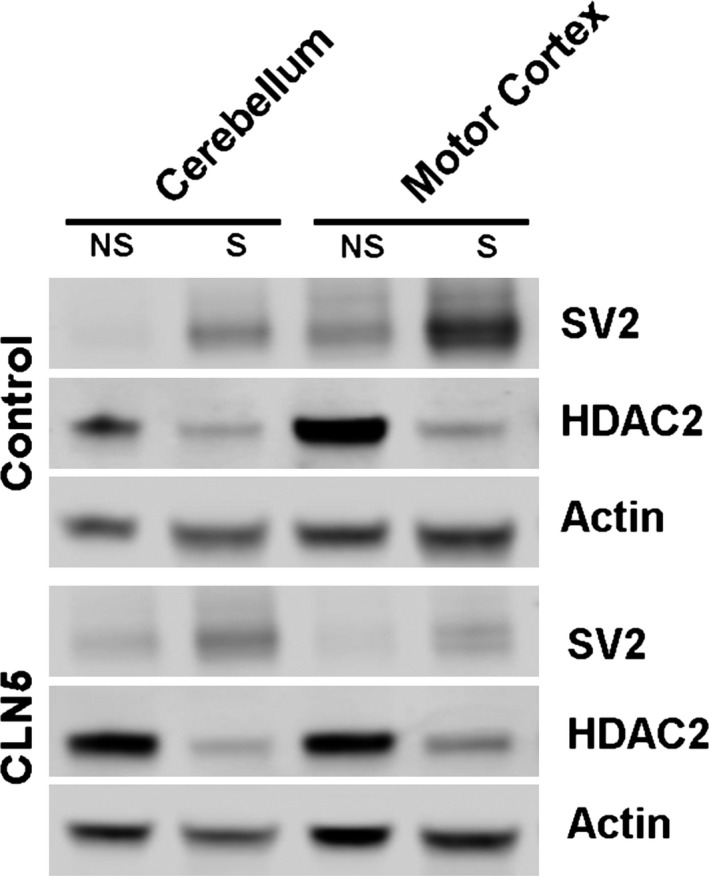
Characterization of synaptosome preparations from control and CLN5 Batten disease sheep brains. Representative fluorescent Western blots showing levels of a core synaptic protein (SV2), a core nuclear protein (HDAC2) and a loading control (actin) in synaptic (S) and nonsynaptic (NS) fractions generated from the cerebellum and motor cortex of control and CLN5 Batten disease sheep brains. Note the enrichment of synaptic protein and exclusion of nuclear protein in the synaptic fractions.

Next, we wanted to establish whether we could generate tissue samples enriched with synaptic material (synaptosomes) from the brains of CLN5 Batten sheep, with a similar efficiency and reproducibility previously found when using mouse tissue (c.f. Wishart et al. 2012; 2014). We adapted the subcellular fractionation protocol previously used for fresh mouse brain for frozen brain samples from the sheep. Larger volumes of buffer and a higher number of passages were required to homogenize sheep tissue when compared to the mouse whole brain. Fluorescent Western blotting on synaptosome preparations confirmed a robust enrichment of a marker synaptic protein, SV2, and almost complete exclusion of a nuclear protein, HDAC2, in synaptosome preparations from both control and CLN5 sheep brains (Fig. 5). Similarly, we found no major differences between synaptosome preparations from the different brain regions, preparations from both motor cortex and cerebellum showing similar levels of enrichment (Fig. 5). Thus, it was possible to use standard synaptosome protocols to generate preparations of synaptically enriched tissue from sheep brains, even using samples that had been frozen and stored.

### Conservation of synaptic proteins between mouse and sheep synaptosomes

**Figure 6:**
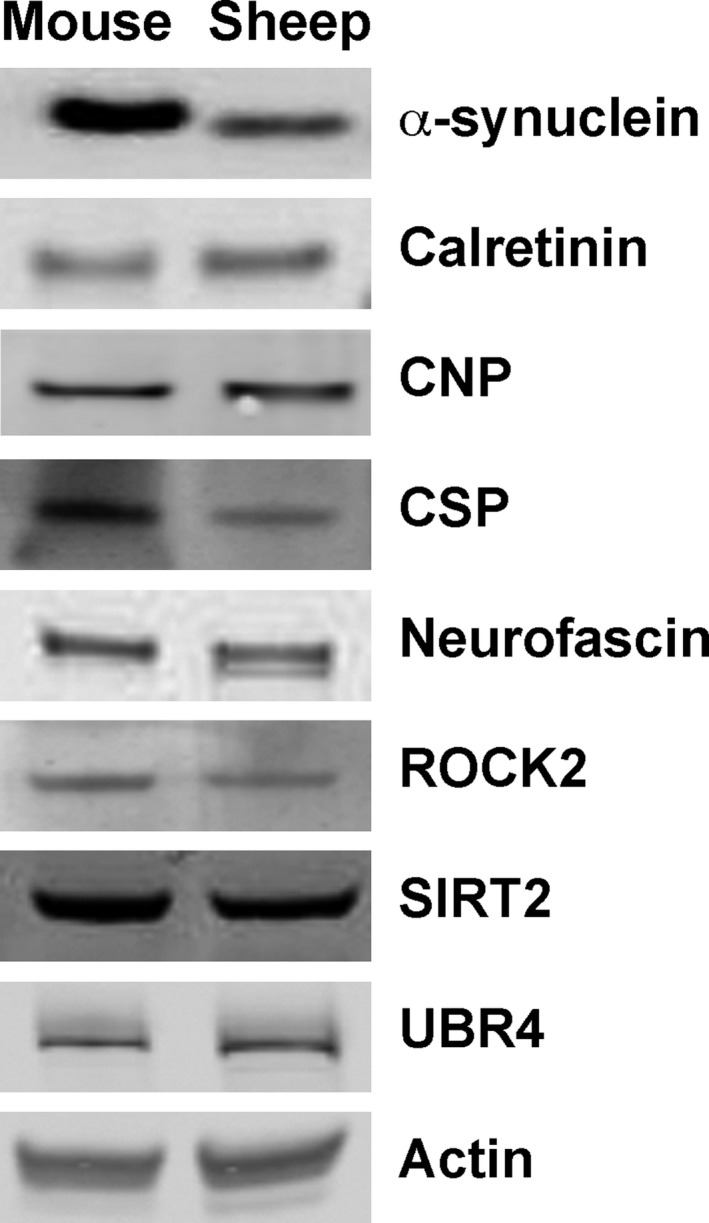
Conserved expression and Western blot detection of synaptic proteins in synaptosome preparations from mice and sheep. Representative fluorescent Western blots showing levels of eight synaptic proteins implicated in the regulation of synaptic stability and degeneration, as well as a loading control (actin), in synaptosome preparations from mouse and sheep brain. Note how all synaptic proteins previously identified in mouse synapses were similarly present, and detectable using the same primary antibodies, in sheep synapses.

Our success in isolating synaptosome preparations from the cerebellum and motor cortex of sheep brains allowed us to next ask whether known molecular regulators of synaptic pathophysiology, previously identified in *Drosophila* and mouse models, were similarly present in synapses from the sheep brain, and could be identified using primary antibodies previously used for studies of the mouse nervous system (Wishart et al. 2012). In these experiments, we compared levels of eight individual proteins previously shown to be present in mouse synapses, and whose expression levels were robustly modified in synapses undergoing degeneration: *α*‐synuclein, calretinin (Calb2), 2′,3′‐cyclic nucleotide 3′ phosphodiesterase (CNP), cysteine‐string protein alpha (CSP‐*α*; DNAJC5), neurofascin, rho‐associated, coiled‐coil containing protein kinase 2 (ROCK2), sirtuin 2 (SIRT2), and ubiquitin protein ligase E3 component n‐recognin 4 (UBR4) (Wishart et al. 2012). Direct comparison of these proteins in synaptosome preparations from mice and sheep, using fluorescent Western blotting, confirmed that all eight proteins were conserved between the two species and were detectable using the same primary antibodies (Fig. 6).

### Region‐specific modifications in known regulators of synaptic stability in CLN5 Batten sheep

Our ability to detect and measure levels of individual proteins implicated in the regulation of synaptic degeneration (Fig. 6) allowed us to directly test whether molecular pathways being instigated in degenerating synapses from the sheep brain are the same as those previously reported in the mouse brain. We therefore generated region‐specific synaptosome preparations from the brains of five control and eight CLN5 Batten disease sheep. Quantitative fluorescent Western blotting was used to measure and compare levels of the eight synaptic proteins previously found to be conserved in the sheep brain.

**Figure 7:**
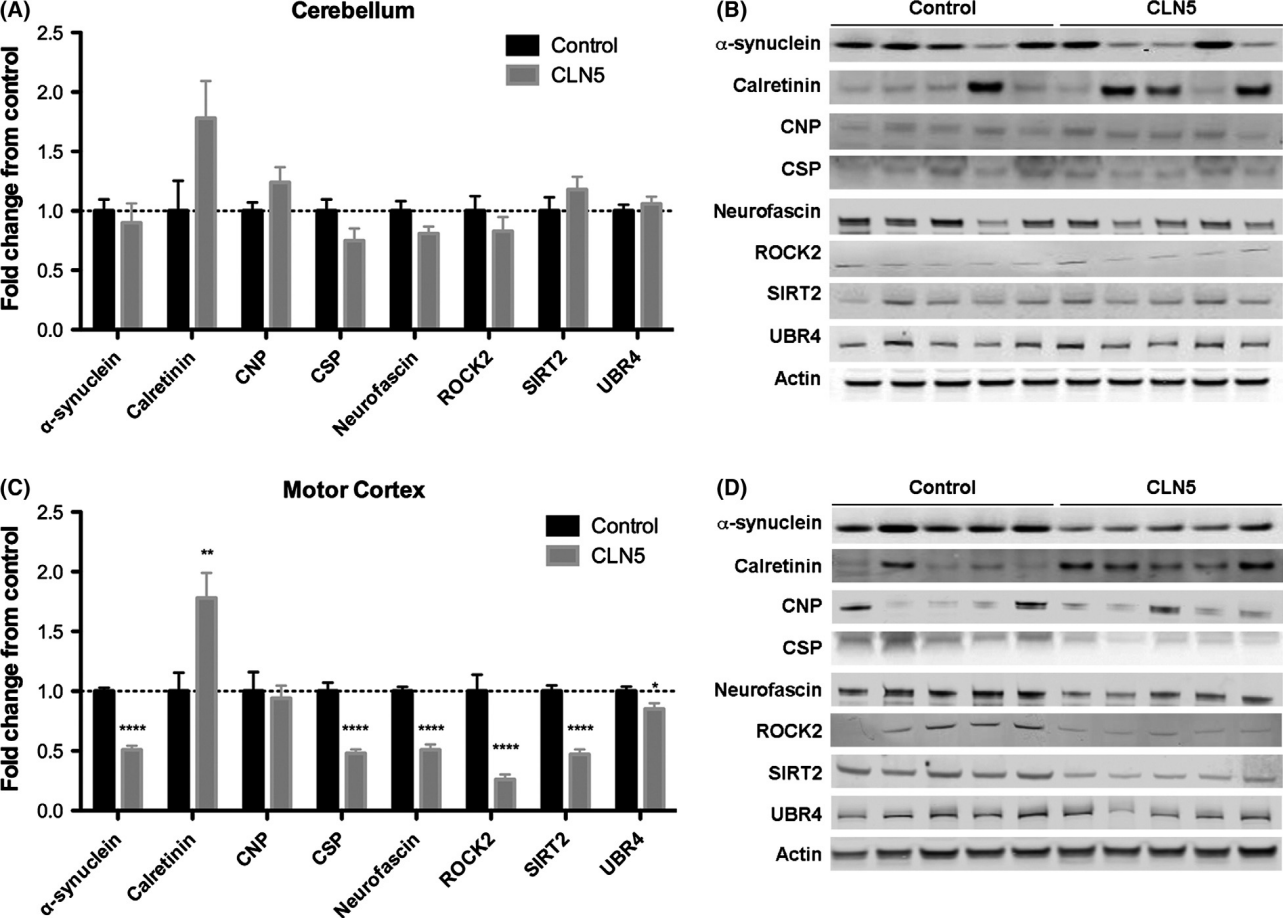
Modified expression levels of synaptic proteins in motor cortex from CLN5 Batten disease sheep. A–B. Levels of the eight synaptic “degeneration” proteins (actin was used as a loading control) in synaptosome preparations generated from the unaffected cerebellum of control and CLN5 Batten disease sheep are shown. Data in panel A (*N* = 5, *n* = 10 control; *N* = 8, *n* = 16 CLN5) are shown as mean ± SEM. Statistical analyses revealed no significant differences (*P* > 0.05 in *t*‐test) in the level of any protein between control and CLN5 samples. Representative Western blots from a single experimental run (*N* = 5 animals per genotype) are shown in panel B. C–D. Levels of the eight synaptic “degeneration” proteins (actin was used as a loading control) in synaptosome preparations generated from the affected motor cortex of control and CLN5 Batten sheep are shown. Data in panel C (*N* = 5, *n* = 10 control; *N* = 8, *n* = 16 CLN5) are shown as mean ± SEM. Statistical analyses revealed significant differences in the levels of seven proteins between control and CLN5 samples (*P < 0.05; **P < 0.01; ****P < 0.0001; in *t*‐test). Representative Western blots from one experimental run (*N* = 5 animals per genotype) are shown in panel D.

An initial comparison of protein levels in synaptosomes generated from the cerebellum (a minimally affected brain region; see above) revealed no significant differences in expression levels of any of these proteins in cerebella from control and CLN5 sheep (Fig. 7A). This analysis confirmed that we could repeatedly detect and measure all eight synaptic proteins across samples from multiple animals, and also demonstrated that levels of these proteins are relatively stable in areas of the brain from CLN5 Batten disease sheep not grossly affected by neurodegeneration.

In striking contrast, a comparison of protein levels in the motor cortex (a severely affected brain region; see above) revealed significant differences in the expression of seven out of the eight proteins examined (Fig. 7B). Several proteins were reduced to less than half in CLN5 cortical synapses compared to controls (*α*‐synuclein, CSP‐*α*, Neurofascin, ROCK2, and SIRT2). In contrast, there was almost twice as much calretinin in synapses from the affected motor cortex than from control sheep. Thus, synaptic proteins previously implicated in synaptic degeneration pathways in mice and *Drosophila* also presented dynamic modifications to their expression levels in synapses from neurodegenerating regions from CLN5 Batten disease sheep compared to controls.

## Discussion

This study set out to address whether cellular and molecular aspects of synaptic pathophysiology previously identified in *Drosophila* and mouse models of a range of diseases (including Batten disease), are recapitulated in larger animal models of neurodegenerative disease. Our initial findings confirmed the regionally restricted nature of disruption to synaptic integrity in areas of the brain of CLN5 sheep undergoing significant neurodegeneration (e.g. loss of SV2 was observed in the affected motor cortex but not in the spared cerebellum). Previous studies using mouse and *Drosophila* models of Batten disease have revealed that synaptic disruption is intimately linked to the regional onset of neurodegeneration (Cooper 2010; Bond et al. 2013), with our findings demonstrating that the relationship between synaptic pathology and neurodegeneration is likely to be similarly conserved in CLN5 sheep. However, given the symptomatic nature of the sheep investigated in this study, further studies examining synaptic changes at pre and early‐symptomatic time points will now be required in order to establish whether synaptic loss precedes, or occurs concurrently with, gross neuropathological changes.

Interestingly, in some other forms of Batten disease, the course of glial activation and subsequent neurodegeneration may occur differently in mice compared to large animals and humans (albeit with inherent difficulties when comparing across species and different genetic mutations). For example, cerebellar involvement has been noted in the PPT1‐deficient mouse model of infantile NCL (Macauley et al. 2009), whereas glial activation and neurodegeneration generally begins in the upper cortex in large animal models of Batten disease (best described in CLN6 sheep (Oswald et al. 2005, 2008)) and human Batten disease patients, with the cerebellum remaining largely spared. In contrast, the neuropathological changes we report here suggest similar regional targeting in CLN5 mice (von Schantz et al. 2009) and sheep.

We also demonstrated the ability to generate synaptosome preparations from frozen sheep brain samples, and used this technique to confirm conserved expression of synaptic proteins between sheep and mouse models of disease. Finally, we found that molecular pathways previously found to regulate synaptic pathophysiology in *Drosophila* and mice were reproduced in synapses from affected regions of CLN5 sheep brain. Taken together, these findings suggest that molecular mechanisms underlying synaptic pathophysiology are conserved in an established sheep model of Batten disease, demonstrating the importance of studies utilizing large animal models for the identification, development, and testing of therapies aimed at delaying or halting synaptic pathology across a range of neurodegenerative conditions.

The demonstration of synaptic involvement in neuropathological pathways activated in affected brain regions from CLN5 sheep provides significant additional experimental support for the hypothesis that synapses play an important role in the progression of neurodegeneration in the NCLs (Virmani et al. 2005; Luiro et al. 2006; Partanen et al. 2008; Kielar et al. 2009; Koch et al. 2011). The additional support gained from analyses of this large animal model indicates that the synaptic phenotypes previously reported in a range of in vivo and in vitro studies of NCLs utilizing lower organisms (Virmani et al. 2005; Luiro et al. 2006; Partanen et al. 2008; Kielar et al. 2009; Koch et al. 2011) are a conserved feature of these diseases applicable to all animal models as well as human patients. This finding places the NCLs alongside a range of other neurodegenerative diseases where synaptic pathology is a major focal point for disease instigation and progression (including Alzheimer's disease, Huntington's disease and motor neuron disease (Wishart et al. 2006)). Moreover, the finding that molecular mechanisms underlying synaptic pathophysiology are conserved across several different neurodegenerative conditions (Wishart et al. 2012) suggests that studies examining mechanisms of synaptic pathology in one condition (such as the NCLs) may also have applicability to other related neurodegenerative conditions.

Alongside the finding that cellular aspects of synaptic pathology were conserved between large and small animal models of the NCLs, the demonstration that molecular regulation of synaptic pathophysiology is also conserved between species is likely to be of particular importance for future studies, using animal models to design and test synaptically targeted therapies for humans. For example, levels of synaptic *α*‐synuclein were significantly reduced in the motor cortex, but not the cerebellum, of CLN5 sheep. *α*‐Synuclein is known to regulate synaptic stability and function in a variety of other animal model systems, including mouse neurons (Volpicelli‐Daley et al. 2011; Rockenstein et al. 2014). Changes in *α*‐synuclein have been reported in many studies of Batten disease, particularly in CLN1 and CLN10 (cathepsin D) deficient mice (Cullen et al. 2009; Blom et al. 2013; Koch et al. 2013) and recently in Atp13a2‐deficient (CLN12) mice (Schultheis et al. 2013). *α*‐Synuclein is known to modulate synaptic pathology in mice, at least in part through interactions with another synaptic protein, CSP‐*α* (Chandra et al. 2005). Mutations in *CSP*‐*α* lead to a dominant adult form of NCL, Kuf's disease (CLN4 (Nosková et al. 2011; Benitez et al. 2011)), and levels of CSP‐*α* were also significantly modified in synapses from affected regions of CLN5 sheep brain. Similar links have been established for many of the other proteins we examined in our study, where previous data from a combined mouse/*Drosophila* study identified them as potential regulators of synaptic and axonal degeneration (Wishart et al. 2012). Thus, many of the molecular regulators of synaptic pathophysiology identified in studies using lower organisms appear to be conserved in the sheep model of CLN5 Batten disease, establishing a clear path from small animal to large animal to human patients with neurodegenerative disease.

Given the considerable overlap in the molecular characteristics of synaptic pathology between sheep and small animal models, it follows that therapies designed to target these pathways, shown to be affective in small animal models, could be taken forward into sheep models, better placed to replicate the size‐ and age‐related aspects of the human nervous system (Aigner et al. 2010; Morton and Howland 2013; Pouladi et al. 2013; Dolezalova et al. 2014). Demonstration of therapeutic effectiveness of a given treatment in a large animal model would provide a robust basis for subsequent translation to cohorts of human patients. This approach is likely to reduce the financial waste associated with failed clinical trials based on data from small animal models (Perrin 2014), and speed up the identification of the best candidate therapies for human patients.

## Funding

This work was supported by a CASE studentship from the BBSRC/GSK (to ISA & THG; BB/K500999/1). TMW is currently a Career Track Fellow at the Roslin Institute, supported by BBSRC ISPG funding. The sheep at Lincoln and laboratory work there was supported by grants from the Batten Disease Support and Research Association (BDSRA) and the Neurological Foundation of New Zealand while CHDI *Inc*. (via AJ Morton) funded the export of the animals sent to Cambridge. Funding to support maintaining the animals and harvesting the tissue in Cambridge was provided by a grant from CHDI *Inc*. (as part of a grant to AJ Morton).

## Conflict of Interest

Authors ISA and THG receive grant funding from a CASE Studentship Award partly supported by GlaxoSmithKline.
